# Squamous cell carcinoma of rectum presenting in a man: a case report

**DOI:** 10.1186/1752-1947-4-392

**Published:** 2010-11-30

**Authors:** A Syed Sameer, Nidda Syeed, Nissar A Chowdri, Fazl Q Parray, Mushtaq A Siddiqi

**Affiliations:** 1 Department of Immunology and Molecular Medicine, Sher-I-Kashmir Institute of Medical Sciences, Soura, Srinagar, Kashmir, 90011, India; 2 Department of Clinical Biochemistry, Sher-I-Kashmir Institute of Medical Sciences, Soura, Srinagar, Kashmir, 190011, India; 3 Department of General Surgery, Sher-I-Kashmir Institute of Medical Sciences, Soura, Srinagar, Kashmir, 190011, India

## Abstract

**Background:**

Primary squamous cell carcinomas of the colorectum are very uncommon. Until now, to the best of our knowledge, only 114 cases of squamous cell carcinoma in the colorectum exist in the reported literature. Here we report a case of squamous cell carcinoma of the rectum in the ethnic Kashmiri population in northern India.

**Case Presentation:**

The case of a 60-year-old male patient (Asian) with a pure squamous cell carcinoma of the rectum is presented here. The patient underwent a curative surgery with concomitant chemotherapy. Two years after the initial curative resection of the tumor he is still alive.

**Conclusion:**

The prognosis for squamous cell carcinoma of the colorectum is worse than for that of adenocarcinoma, because of the delayed diagnosis. The etiopathogenicity of squamous cell carcinoma of the colorectum is discussed. Surgical resection of the lesion seems to be the treatment of choice. Chemotherapy also helps in improvement of the prognosis.

## Introduction

The occurrence of squamous cell carcinomas (SCC) in the colorectum is a rare entity representing a small fraction of colorectal malignancies, since more than 90% of colorectal diseases are adenocarcinoid tumors [1]. Very little information is available in the literature about the etiology, prognosis and optimal treatment of this malignancy [2]. Here in this study, we describe a patient with SCC of the rectum who underwent a lower anterior resection (LAR) for the possible treatment of the malignancy.

## Case presentation

**Figure 1:**
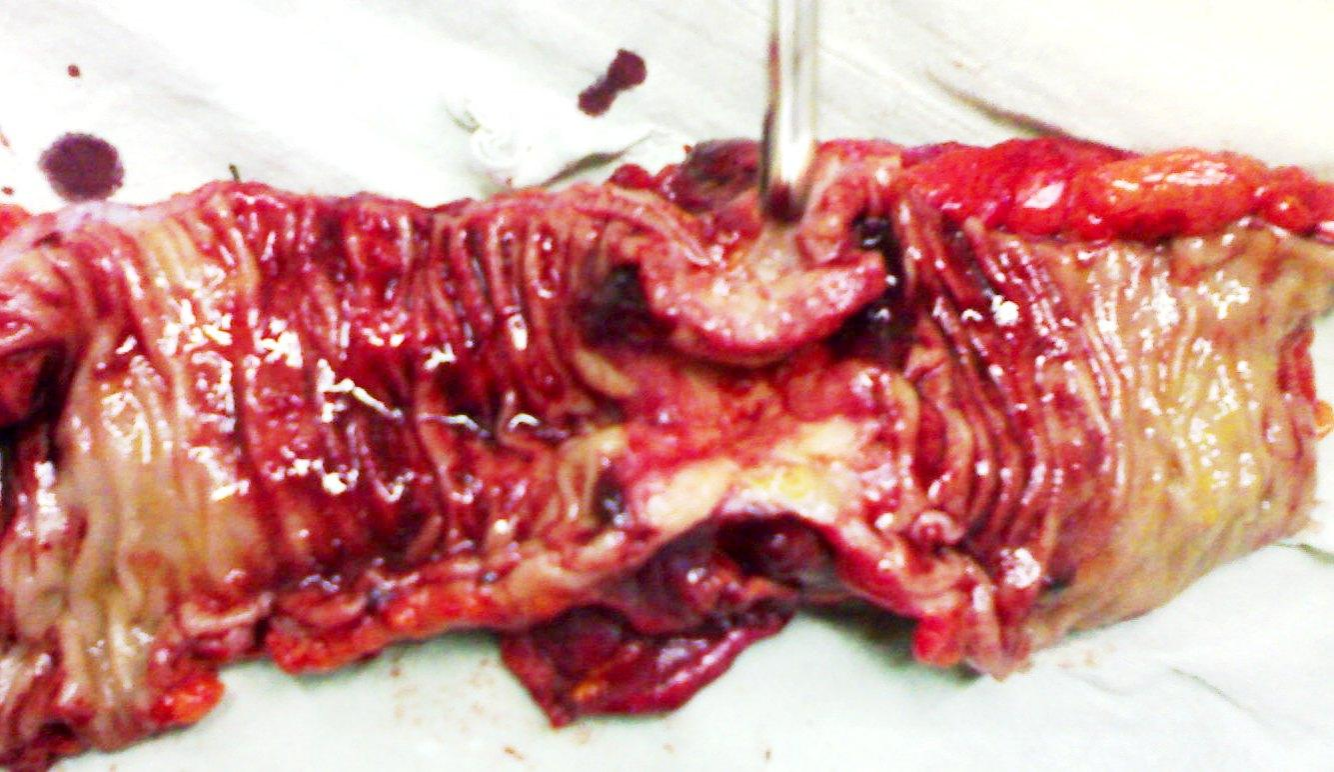
**Image showing the inner lining of the colon with a rosette-shaped malignant tumor at the lateral wall of the rectum**.

A 60-year-old male patient from an urban area of Kashmir (Asian) visited the Department of General Medicine of our institute with the chief complaints of severe lower-abdominal pain for the past eight months. The patient also complained of severe constipation, nausea, vomiting, anorexia, loss of appetite, abdominal cramps, incontinence of faeces and weight loss during the past four months. He experienced profuse bleeding from the rectum for the last month. Initial interviews with the patient revealed that the he was a heavy smoker and frequent user of *noon-chai *(Salt tea), meat and pickles. On examination the patient was found to be anemic. Digital rectal examination revealed an ulcero-infiltrative lesion with restricted mobility about 4 cm from the anal verge on the left lateral wall. A colonoscopy confirmed the rectal examination and biopsies taken at the time of the colonoscopy revealed squamous cell carcinoma (SCC) of basal cell type in the first histopathological examination. The report was re-confirmed by a second independent pathologist. A Contrast-Enhanced Computed Tomography (CECT) of the chest, abdomen and pelvis was also done but no lesions were found in any other site than the rectum. The lesion was without any fat stranding or lymphadenopathy. Furthermore, following the provisional diagnosis, the patient was referred to the Department of General Surgery for radical treatment, where he underwent LAR of the rectum using the standard technique of mesorectal excision (Figure 1). The continuity of the gut was restored by a circular stapler for low colorectal anastomosis with formation of a colonic pouch. The colonic pouch takes over the function of rectal reservoir which is lost after excision of the middle and lower rectum. Microscopic examination of the resected lesion demonstrated a 2.5 cm × 3 cm SCC tumor of the rectum infiltrating the serosa. The margins of the excised tissue were found to be free of the tumor. However, four regional lymph nodes were also infiltrated by the metastatic SCC cells. The liver and the rest of the organs were free of any metastasis. The slides were reviewed by a third histopathologist who reported the lesion as poorly differentiated squamous cell carcinoma. The stage of the tumor was found to be T_3_N_2_M_o_. The post-operative period was uneventful. Post-operatively the patient received four cycles of chemotherapy with cisplastin and 5-fluorouracil for five days. The patient is on two years of follow-up and has not shown any evidence of recurrenceas of the present time.

## Discussion

Colorectal cancer (CRC) is the third most common cause of cancer-related death in the world [3]. Almost 90% of CRC are adenocarcinomas, while the remaining 10% are made up of carcinomas, sarcomas and lymphoid tumors [1]. The occurrence of SCC in the gastrointestinal tract (GIT) is a rare phenomenon, and its occurrence in the colorectum is extremely unusual [4]. The incidence of SCC of the colorectum has been reported to be almost 0.1 to 0.25 per 1000 CRC [4,5]. A look into the research work and the reported cases of SCC dates back to 1907, when Herxheimer reported adenosquamous carcinoma of the cecum but it was in 1919 when the first case of pure SCC of the colon was reported by Schmidtmann [6] in a 65-year-old man [7]. It was not until 1933 that the first case involving the rectum was subsequently described by Raiford [8]. In India, Bhat *et al*. [9] reported the first case of pure SCC of the colon in 1993 in a 55-year-old female from the southern part of the country. Until now almost 120 cases of SCC have been reported from all over the world (See Table 1). Surprisingly, a study from Russia reported 107 cases of SCC from a single center alone [10] but there has been no such reports of high incidence of SCC in the colorectum from any other part of the world.

**Table 1:** Reported cases of squamous cell carcinoma of the colorectum (Data available from 1933 to 2009)

Study number	Study	Age	Sex	Surgery	Outcome
01.	Schmidtmann (1919) [6]	65	M	NA	Died after 1 m

02.	Raiford (1933) [8]	43	F	NA	Died after 7 m

03.	Catell *et al*. (1943) [22]	63	M	LAR	Alive at 3.5 y

04.	Wiener *et al*. (1962) [23]	52	F	APR	Died at 1 y

05.	Larizaden and Powell (1965) [24]	44	F	APR	Died at 1 y

06.	Cabrera *et al*. (1967) [25]	62	F	APR	NR
		
		50	F		NR

07.	Minkowitz *et al*. (1967) [26]	49	F	Proctocolectomy	Died after 5 m

08.	Gaston *et al*. (1967) [27]	65	M	Hemicolectomy	Alive at 2 y

09.	Pemberton and Lendrum (1968) [28]	48	F	Hemicolectomy	Alive at 2 y

10.	Birnbaum *et al*. (1970) [29]	82	M	Hemicolectomy	NR

11.	Corner *et al*. (1971) [14]	34	F	APR	Alive at 13 y

12.	Lewis *et al*. (1971) [30]	61	M	Hemicolectomy	Died after 10 d

13.	Balfour (1972) [31]	63	M	NA	Died after 18 m

14.	Horne and McCulloch (1978) [32]	53	M	Hemicolectomy	Died after 11 m

15.	Crissmann (1978) [33]	72	M	Colectomy	Died after 3 d

16.	Burgess *et al*. (1979) [34]	43	M	Hemicolectomy	Died after 11 m

17.	Williams *et al*. (1979) [11]	45	M	APR	Died after 9 m

18.	Lasser *et al*. (1980) [35]	65	F	N/A	Alive at 3 y
		
		48	F	N/A	Alive 8 m
		
		54	M	N/A	Alive 17 m

19.	Hickey and Corson (1981) [36]	48	F	Hemicolectomy	Alive at 21 m

20.	Petrelli *et al*. (1981) [37]	73	M	Colectomy	Died after 9 d

21.	Pitella and Torres (1982) [38]	33	M	Ileocolic bypass	Died after 10 d

22.	Hey and Brandt (1982) [39]	NA	NA	NA	NA
		
		NA	NA	NA	NA

23.	Lyttle *et al*. (1983) [40]	65	F	Hemicolectomy	Alive at 2 m

24.	Vezeridis *et al*. (1983) [41]	56	M	APR	Died after 10 m
		
		44	M	APR	Died after 9 d
		
		61	F		Died after 4 m
		
		66	F		Died after 15 m
		
		62	F	APR	Died after 13 m

25.	Gould *et al*. (1983) [42]	61	M	Ileocolic bypass	Died after 3 m

26.	Francioni *et al*. (1983) [43]	NA	NA	NA	NA

27.	Forouhar *et al*. (1984) [44]	NA	NA	NA	NA

28.	Lafreniere *et al*. (1985) [13]	60	M	TAE	Alive at 2 y

29.	Balsano *et al*. (1985) [45]	65	M	Hemicolectomy	NA
		
		58	M	Hemicolectomy	NA

30.	Chulia et al. **(1986**) [46]	NA	NA	NA	NA

31.	Weidner and Zekan, (1986) [47]	73	M	NA	Died after 4 y

32.	Piggott and Williams (1987) [48]	60	F	APR	Alive at 13 m

33.	Woods *et al*. (1987) [49]	57	F	APR	Died after 3 m

34.	Shao *et al*. (1987) [50]	NA	NA	NA	NA

35.	Prener *et al*. (1988) [51]	43	F	APR	Died after 1 y
		
		77	F	Polypectomy	Died after 3 y
		
		55	F	APR	Alive at 3 y
		
		55	M	APR	Died after 3 m
		
		53	M	APR	Died after 1 y

36.	Lundquest *et al*. (1988) [52]	NA	NA	NA	NA

37.	Wyatt (1991) [53]	71	M	NA	Alive at 1 y

38.	Schneider *et al*. (1992) [54]	44	M		NA
		
		69	F	TAE	Alive at 3 y

39.	Betancourt *et al*. (1992) [55]	NA	NA	NA	NA

40.	Vignale *et al*. (1993) [56]	69	M	NA	NA

41.	Yoshida *et al*. (1994) [57]	51	M	Hemicolectomy	Died after 39 d

42.	Vraux *et al*. (1994) [58]	NA	NA	Chemotherapy	Died after 5 y

43.	Alekseev *et al*. (1994) [59]	NA	NA	NA	NA

44.	Petrelli *et al*. (1996) [60]	62	M	APR	NA
		
		41	F	Colectomy	NA

45.	Martinez-Gonzalez *et al*. (1996) [61]	40	M	LAR	Alive at 18 m

46.	Juturi *et al*. (1998) [62]	61	F	Hemicolectomy	Alive at 18 y
	
		61	M	Hemicolectomy	Died after 15 m

47.	Kim *et al*. (2001) [63]	41	F	LAR	Died after 4 m

48.	Copur *et al*. (2001) [64]	54	M	APR+CT	NA

49.	Sotlar *et al*. (2001) [65]	87	M	LAR	Died after 20 m

50.	Frizelle *et al*. (2001) [66]	9 cases			

51.	Gelas *et al*. (2002) [2]	47	F	APR+CT	Alive at 16 y
		
		63	M	APR+CT	Died after 14 m
		
		70	F	APR	Died after 18 m
		
		93	M		Died after 4 m
		
		45	F	LAR	Alive at 6 m
		
		43	F	LAR	Alive at 2 y

52.	Bhat *et al*. (2003) [9]	55	F	Hemicolectomy	NA

53	Kim, 2005 [67]	71	M		NA

54.	Anagnostopoulos *et al*. (2005) [7]	75	M	APR	Alive at 14 m

55.	Lam *et al*. (2006) [68]	44	F	LAR	NA

56.	Theodosopoulos *et al*. (2006) [21]	39	F	APR	Alive at 18 m

57.	Ambrosini-Spaltro *et al*. (2006) [69]	81	M	Hemicolectomy	Alive at 2 y

58.	Pikarsky *et al*. (2006) [70]	57	F		Alive at 7 yr

59.	Nahas *et al*. (2007) [5]	58	F10/M2		Alive at 2.6 yr

60.	Miyamoto (2007) [1]	89	M	Colectomy	Died after 3 m

61.	Cheng *et al*. (2007) [71]	51	F	Proctocolectomy	NA

62.	Kong *et al*. (2007) [72]	48	F	TAE	Alive at 3 y

		53	F	NA	

63.	Clark *et al*. (2008) [73]	75	M		Alive at 20 m
		
		71	F		Alive at 31 m
		
		42	F		Alive at 13 m
		
		70	M		Alive at 14 m
		
		55	F	LAR	Alive at 19 m
		
		45	F		Alive at 23 m
		
		71	F		Alive at 5 m

64.	Rasheed *et al*. (2009) [74]	55	F		Alive at 11 y
		
		50	M		Alive at 7 y
		
		69	F		Alive at 4 y
		
		61	M	APR	Alive at 4 y
		
		58	M	APR	Alive at 2 y
		
		41	F		Alive at 2 y

65.	Our Case	60	M	LAR	Alive at 15 m

NA: not available; F: female; M: male; LAR: low anterior resection; APR: abdominoperineal resection; TAE:transanal excision; y: years; m: months and d: days

Before the diagnosis of primary SCC of colorectum is made, certain criteria must be fulfilled as given by Williams *et al*. in 1979 [11]. This criteria includes: (A) absence of evidence of squamous cell carcinoma of any other part of the body, ruling out any chance of possible metastasis from any organ to the colorectal site; (B) exclusion of any proximal extension of anal squamous cell carcinoma; (C) absence of fistulous tract lined by squamous cells; and (D) confirmation of SCC by histological analysis [1,4,12]. All of these criteria were fulfilled by our case.

A look at the available literature reveals that squamous cell carcinoma of the colorectum affects individuals with a mean age of 55 to 60 years Women are more frequently predisposed to SCC than men, around 66% of cases occurred in women and 34% in men. Furthermore, SCC occurs in concomitance with an advanced tumor stage (Duke's C) [4,13]. Since SCC of the rectum is a rare tumor, epidemiological data constituting patient demographics, risk factors and natural history are lacking in the literature. The clinical characteristics of the patients with SCC of the colorectum are similar to those with adenocarcinoma: rectal bleeding, abdominal pain, change in bowel habits and weight loss [4]. Because of the rare nature of this malignancy the prognosis for patients is difficult to establish, Comer *et al*. suggested a poorer prognosis for patients with colorectal SCC than adenocarcinoma [1,4,14].

Almost four different pathophysiological theories regarding the origin of squamous cell carcinoma of the colorectum have been proposed in the literature so far. These can be summarized as: (A) Proliferation of uncommitted basal cells into squamous cells which undergo malignant transformation following mucosal injury [15]; (B) Ability of pluripotent stem cells to undergo spontaneous squamous differentiation [16]; (C) Squamous metaplasia of glandular epithelium resulting from chronic inflammation or irritation, secondary to inflammatory bowel disease [17], infection [18] or radiation [19]; (D) Origin from embryonal nests of ectodermal cells; and (E) Arousal of carcinomas from preexisting adenomas or adenocarcinomas [7,20].

## Conclusion

In conclusion, advanced colorectal SCC has a poor prognosis. Since colorectal SCC is a very rare disease, treatment selection is difficult. However, surgical resection and adjuvant chemotherapy [21] is a better approach to the treatment of colorectal SCC.

## Consent

Written informed consent was obtained from the patient for publication of this case report and accompanying images. A copy of the written consent is available with the corresponding author of this manuscript and is accessible for review by the Editor-in-Chief of this journal

## Competing interests

The authors declare that they have no competing interests.

## Authors' contributions

ASS conceived and designed the study and wrote the manuscript. NS suggested the necessary changes and copyedited the manuscript. NAC and FQP procured and provided the tumor samples for the study. MAS coordinated the study and revised the manuscript. All authors read and approved the final manuscript.

## References

[B1] Miyamotoa H Nishiokaa M Kuritaa N Hondaa J Yoshikawaa K Higashijimaa J Miyatania T Bandoub Y Shimadaa M Squamous cell carcinoma of the descending colon: report of a case and literature review Case Rep Gastroenterol 2007 1 77 83 10.1159/000107470 PMC3073792 21487550

[B2] Gelas T Peyrat P Francois Y Gerard JP Baulieux J Gilly FN Vignal J Glehen O Primary squamous-cell carcinoma of the rectum: report of six cases and review of the literature Dis Colon Rectum 2002 45 1535 1540 10.1007/s10350-004-6462-z 12432303

[B3] Sameer AS Rehman S Pandith AA Syeed N Shah ZA Chowdhri NA Wani KA Siddiqi MA Molecular gate keepers succumb to gene aberrations in colorectal cancer in Kashmiri population, revealing a high incidence area Saudi J Gastroenterol 2009 15 244 252 10.4103/1319-3767.56102 19794270 PMC2981841

[B4] Dyson T Draganov PV Squamous cell cancer of the rectum World J Gastroenterol 2009 15 4380 4386 10.3748/wjg.15.4380 19764088 PMC2747057

[B5] Nahas CS Shia J Joseph R Schrag D Minsky BD Weiser MR Guillem JG Paty PB Klimstra DS Tang LH Wong WD Temple LK Squamous-cell carcinoma of the rectum: a rare but curable tumor Dis Colon Rectum 2007 50 1393 1400 10.1007/s10350-007-0256-z 17661147

[B6] Schmidtmann M Zur Kenntnis seltener Krebsformen Virchow Arch (A) 1919 226 100 118 10.1007/BF02039541

[B7] Anagnostopoulos G Sakorafas GH Kostopoulos P Grigoriadis K Pavlakis G Margantinis G Vugiouklakis D Arvanitidis D Squamous cell carcinoma of the rectum: a case report and review of the literature Eur J Cancer Care Engl 2005 14 70 74 10.1111/j.1365-2354.2005.00523.x 15698388

[B8] Raiford TS Epitheliomata of the lower rectum and anus Surg Gynecol Obstet 1933 57 21 35

[B9] Bhat S Pai M Premnath RP Primary squamous cell carcinoma of caecum Indian J Cancer 2003 40 118 119 14716117

[B10] Mel'nikov RA Goshchitskiĭ LG Kovalev VK Clinical manifestations of squamous cell carcinoma of the rectum Vopr Onkol 1984 30 76 83 6485288

[B11] Williams GT Blackshaw AJ Morson BC Squamous carcinoma of the colorectum and its genesis J Pathol 1979 129 139 147 10.1002/path.1711290306 529012

[B12] Carroll D Rajesh PB Colonic metastases from primary squamous cell carcinoma of the lung Eur J Cardiothorac Surg 2001 19 719 720 10.1016/S1010-7940(01)00646-7 11343961

[B13] Lafreniere R Ketcham AS Primary squamous carcinoma of the rectum. Report of a case and review of the literature Dis Colon Rectum 1985 28 967 972 10.1007/BF02554319 4064861

[B14] Comer TP Beahrs OH Dockerty MB Primary squamous cell carcinoma and adenoacanthoma of the colon Cancer 1971 58 111 117 10.1002/1097-0142(1971)28:5<1111::aid-cncr2820280504>3.0.co;2-v 5125659

[B15] Michelassi F Montag AG Block GE Adenosquamous-cell carcinoma in ulcerative colitis. Report of a case. Dis Colon Rectum 1988 31 323 326 3282843 10.1007/BF02554371

[B16] Ouban A Nawab RA Coppola D Diagnostic and pathogenetic implications of colorectal carcinomas with multidirectional differentiation: a report of 4 cases Clin Colorectal Cancer 2002 1 243 248 10.3816/CCC.2002.n.006 12450423

[B17] Fu K Tsujinaka Y Hamahata Y Matsuo K Tsutsumi O Squamous metaplasia of the rectum associated with ulcerative colitis diagnosed using narrow-band imaging Endoscopy 2008 40 E45 E46 10.1055/s-2007-966861 18300203

[B18] Audeau A Han HW Johnston MJ Whitehead MW Frizelle FA Does human papilloma virus have a role in squamous cell carcinoma of the colon and upper rectum? Eur J Surg Oncol 2002 28 657 660 10.1053/ejso.2002.1304 12359204

[B19] Yurdakul G de Reijke TM Blank LE Rauws EA Rectal squamous cell carcinoma 11 years after brachytherapy for carcinoma of the prostate J Urol 2003 169 280 10.1016/S0022-5347(05)64092-X 12478160

[B20] Jaworski RC Biankin SA Baird PJ Squamous cell carcinoma in situ arising in inflammatory cloacogenic polyps: report of two cases with PCR analysis for HPV DNA Pathology 2001 33 312 314 10.1080/00313020120062901 11523931

[B21] Theodosopoulos TK Marinis AD Dafnios NA Vassiliou JG Samanides LD Carvounis EE Smyrniotis VE Aggressive treatment of metastatic squamous cell carcinoma of the rectum to the liver: a case report and a brief review of the literature World Journal of Surgical Oncology 2006 4 49 10.1186/1477-7819-4-49 16895595 PMC1555584

[B22] Catell RB Williams AG Epidermoid carcinoma of the anus and rectum Arch Surg 1943 46 336 349

[B23] Wiener MF Polayes SH Yidi R Squamous carcinoma with schistosomiasis of the colon Am J Gastroent 1962 37 48 54 14006818

[B24] Larizaden R Powell DE Neoplastic change in a duplicated colon Br J Surg 1965 52 666 668 10.1002/bjs.1800520907 14338313

[B25] Cabrera A Pickren JW Squamous metaplasia and squamous-cell carcinoma of the rectosigmoid Dis Colon Rectum 1967 10 288 297 10.1007/BF02617142 6037409

[B26] Minkowitz S Primary squamous cell carcinoma of the rectosigmoid portion of the colon Arch Pathol 1967 84 77 80 6027742

[B27] Gaston EA Squamous-cell carcinoma of the colon and rectum: report of a case Dis Colon Rectum 1967 10 435 434 10.1002/path.1711290306 6066367

[B28] Pemberton M Lendrum J Squamous-cell carcinoma of the caecum following ovarian adenocarcinoma Br J Surg 1968 55 273 276 10.1002/bjs.1800550409 5644391

[B29] Birnbaum W Squamous cell carcinoma and adenoacanthoma of the colon JAMA 1970 212 1511 1513 10.1001/jama.212.9.1511 5467545

[B30] Lewis PL Harrer WV Sencindiver PV Primary squamous-cell carcinoma of the cecum: report of a case Dis Colon Rectum 1971 14 213 217 10.1007/BF02553188 5139957

[B31] Balfour TW Does squamous carcinoma of the colon exist? Br J Surg 1972 59 410 412 10.1002/bjs.1800590523 5021150

[B32] Horne BD McCulloch CF Squamous cell carcinoma of the cecum: a case report Cancer 1978 42 1879 1882 10.1002/1097-0142(197810)42:4<1879::AID-CNCR2820420427>3.0.CO;2-4 361214

[B33] Crissman JD Adenosquamous and squamous cell carcinoma of the colon Am J Surg Pathol 1978 2 47 54 10.1097/00000478-197803000-00006 637188

[B34] Burgess PA Lupton EW Talbot IC Squamous-cell carcinoma of the proximal colon: report of a case and review of the literature Dis Colon Rectum 1979 22 241244 10.1007/BF02586884 467177

[B35] Lasser P Elias D Eschwege F A propos de 3 cas d'epitheliomas epidermoides du rectum J Chir 1980 117 377 380 7419633

[B36] Hickey WF Corson JM Squamous cell carcinoma arising in a duplication of the colon: case report and literature review of squamous cell carcinoma of the colon and of malignancy complicating colonic duplication Cancer 1981 47 602 609 10.1002/1097-0142(19810201)47:3<602::AID-CNCR2820470330>3.0.CO;2-8 7226009

[B37] Petrelli M Tetangco E Reid JD Carcinoma of the colon with undifferentiated, carcinoid, and squamous cell features Am J Clin Pathol 1981 75 581 584 6261578 10.1093/ajcp/75.4.581

[B38] Pittella JE Torres AV: Primary squamous-cell carcinoma of the cecum and ascending colon: report of a case and review of the literature Dis Colon Rectum 1982 25 483 487 10.1007/BF02553663 7094788

[B39] Hey A Brandt G [A pure squamous cell carcinoma of the large intestine. Report of 3 personal observations and a literature review] Pathologe 1982 3 359 364 7156057

[B40] Lyttle JA Primary squamous carcinoma of the proximal large bowel. Report of a case and review of the literature Dis Colon Rectum 1983 26 279 282 10.1007/BF02562498 6839899

[B41] Vezeridis MP Herrera LO Lopez GE Ledesma EJ Mittleman A Squamous-cell carcinoma of the colon and rectum Dis Colon Rectum 1983 26 188 191 10.1007/BF02560169 6825528

[B42] Gould L Shah JM Khedekar RR Burns WA Squamous cell carcinoma of the splenic flexure of the colon Dig Dis Sci 1983 28 918 922 10.1007/BF01317044 6352206

[B43] Francioni G Canuti S Cardelli A Montesi M [Epidermoid carcinoma of the colon. Clinical case of double recto-sigmoid basalioma] Minerva Dietol Gastroenterol 1983 29 33 38 6843855

[B44] Forouhar F Neoplastic colonic polyp with extensive squamous metaplasia. Case report Tumori 1984 70 99 103 6710610 10.1177/030089168407000116

[B45] Balsano NA Squamous cell carcinoma of the cecum Arch Surg 1985 120 1176 1177 4038062 10.1001/archsurg.1985.01390340072014

[B46] Chulia F Camps C Rodriguez A Medina E Tuset J Epidermoid carcinoma of the colon. Description of a lesion located in the hepatic flexure Dis Colon Rectum 1986 29 665 667 10.1007/BF02560334 3757710

[B47] Weidner N Zekan P Carcinosarcoma of the colon - Report of a unique case with light and immuoistochemical studies Cancer 1986 58 1126 1130 10.1002/1097-0142(19860901)58:5<1126::AID-CNCR2820580525>3.0.CO;2-Q 2425931

[B48] Piggott JP Williams GB Primary squamous cell carcinoma of the colorectum: case report and literature review of a rare entity J Surg Oncol 1987 35 117 119 10.1002/jso.2930350211 3586681

[B49] Woods WG Squamous cell carcinoma of the rectum arising in an area of squamous metaplasia Eur J Surg Oncol 1987 13 455 458 3666162

[B50] Shao YF Pan GL Zhou CN Yu HT: [Squamous cell carcinoma of the ascending colon--a case report and review of literature] Zhonghua Zhong Liu Za Zhi 1987 9 315 316 3315530

[B51] Prener A Nielsen K Primary squamous cell carcinoma of the rectum in Denmark APMIS 1988 96 839 844 10.1111/j.1699-0463.1988.tb00951.x 3166810

[B52] Lundquest DE Marcus JN Thorson AG Massop D: Primary squamous cell carcinoma of the colon arising in a villous adenoma Hum Pathol 1988 19 362 364 10.1016/S0046-8177(88)80532-X 3278968

[B53] Wyatt MG Clarke TJ Teasdale C Primary squamous cell carcinoma of the caecum Eur J Surg Oncol 1991 17 392 394 1845295

[B54] Schneider TA Birkett DH Vernava AM Primary adenosquamous and squamous cell carcinoma of the colon and rectum Int J Colorectal Dis 1992 7 144 147 10.1007/BF00360355 1402312

[B55] Betancourt C Berríos G Peña E: [Squamous cell carcinoma of the colon. A case report] G E N 1992 46 331 335 1340840

[B56] Vignale R Espasandin J Deneo H Gonzalez V: Halo seborrheic keratosis associated with colon carcinoma Int J Dermatol 1993 32 846 10.1111/j.1365-4362.1993.tb02784.x 8270353

[B57] Yoshida J Tohma H Nagata T Okuzono Y Takahashi M Squamous cell carcinoma of the splenic flexure of the colon: report of a case Surg Today 1994 24 75 79 10.1007/BF01676891 8054782

[B58] Vraux H Kartheuser A Haot J Humblet Y Detry R Dive C Kestens PJ Primary squamous-cell carcinoma of the colon: a case report Acta Chir Belg 1994 94 318 320 7846991

[B59] Alekseev VS Boĭkov VP Pavlov NV Karyshev PB [Squamous cell cancer of the colon with inflammation] 1994 12 Khirurgiia Mosk 58 7897958

[B60] Petrelli NJ Valle AA Weber TK Rodriguez-Bigas M Adenosquamous adenocarcinoma of the colon and rectum Dis Colon Rectum 1996 39 1265 1268 10.1007/BF02055120 8918436

[B61] Martinez-Gonzalez MD Takahashi T Leon-Rodriguez E Gamboa-Dominguez A Lome C Garcia-Blanco MC Bezaury P Moran MA Case report of primary squamous carcinoma of the rectum Rev Invest Clin 1996 48 453 456 9028152

[B62] Juturi JV Francis B Koontz PW Wilkes JD Squamous-cell carcinoma of the colon responsive to combination chemotherapy: report of two cases and review of the literature Dis Colon Rectum 1999 42 102 109 10.1007/BF02235191 10211528

[B63] Kim JH Moon WS Kang MJ Park MJ Lee DG Sarcomatoid carcinoma of the colon: a case report. J Korean Med Sci 2001 16 657 660 10.3346/jkms.2001.16.5.657 PMC3057597 11641539

[B64] Copur S Ledakis P Novinski D Mleczko KL Frankforter S Bolton M Fruehling RM VanWie E Norvell M Muhvic J Squamous cell carcinoma of the colon with an elevated serum squamous cell carcinoma antigen responding to combination chemotherapy Clin Colorectal Cancer 2001 1 55 58 10.3816/CCC.2001.n.006 12445380

[B65] Sotlar K Köveker G Aepinus C Selinka HC Kandolf R Bültmann B Human papillomavirus type 16-associated primary squamous cell carcinoma of the rectum Gastroenterology 2001 120 988 994 10.1053/gast.2001.22523 11231953

[B66] Frizelle FA Hobday KS Batts KP Nelson H Adenosquamous and squamous carcinoma of the colon and upper rectum: a clinical and histopathologic study Dis Colon Rectum 2001 44 341 346 10.1007/BF02234730 11289278

[B67] Kim N Luchs JS Halpern D Davis E Donovan V Weston SR Katz DS Radiology-pathology conference: carcinosarcoma of the colon J Clin Imag 2005 29 259 262 10.1016/j.clinimag.2004.09.002 15967317

[B68] Lam AK Ho YH Primary squamous cell carcinoma of the rectum in a patient on immunosuppressive therapy Pathology 2006 38 74 76 10.1080/00313020500467113 16484015

[B69] Ambrosini-Spaltro A Salvi F Betts CM Frezza GP Piemontese A Del Prete P Baldoni C Foschini MP Viale G Oncocytic modifications in rectal adenocarcinomas after radio and chemotherapy Virchows Arch 2006 448 442 448 10.1007/s00428-005-0137-6 16365727

[B70] Pikarsky AJ Belin B Efron J Woodhouse S Weiss EG Wexner SD Nogueras JJ Squamous cell carcinoma of the rectum in ulcerative colitis: case report and review of the literature Int J Colorectal Dis 2007 22 445 447 10.1007/s00384-006-0110-0 16932927

[B71] Cheng H Sitrin MD Satchidanand SK Novak JM Colonic squamous cell carcinoma in ulcerative colitis: Report of a case and review of the literature Can J Gastroenterol 2007 21 47 50 17225882 10.1155/2007/904081 PMC2656630

[B72] Kong CS Welton ML Longacre TA Role of human papillomavirus in squamous cell metaplasia-dysplasiacarcinoma of the rectum Am J Surg Pathol 2007 31 919 925 10.1097/01.pas.0000213441.86030.fc 17527081

[B73] Clark J Cleator S Goldin R Lowdell C Darzi A Ziprin P Treatment of primary rectal squamous cell carcinoma by primary chemoradiotherapy: should surgery still be considered a standard of care? Eur J Cancer 2008 44 2340 2343 10.1016/j.ejca.2008.07.004 18707873

[B74] Rasheed S Yap T Zia A McDonald PJ Glynne-Jones R Chemo-radiotherapy: an alternative to surgery for squamous cell carcinoma of the rectum--report of six patients and literature review Colorectal Dis 2009 11 191 197 10.1111/j.1463-1318.2008.01560.x 18462236

